# Antibiotics in Veterinary Ophthalmology: Resistance, Stewardship, and Emerging Antibiotic‐Sparing Strategies

**DOI:** 10.1111/vop.70209

**Published:** 2026-06-22

**Authors:** Lionel Sebbag, Oren Pe'er

**Affiliations:** ^1^ Koret School of Veterinary Medicine The Hebrew University of Jerusalem Rehovot Israel

**Keywords:** antibiotic stewardship, antimicrobial resistance, antimicrobial susceptibility testing, keratitis, multidrug resistance, one health

## Abstract

Antimicrobial resistance (AMR) is a growing challenge in veterinary ophthalmology, particularly in cases of bacterial keratitis, where progressive stromal infection can threaten vision and globe integrity within hours to days. This review synthesizes current evidence on pathogen distribution, antimicrobial susceptibility profiles, multidrug resistance (MDR) prevalence, and determinants of nonsusceptibility in veterinary patients, highlighting the emerging role of antibiotic‐sparing alternatives. Across contemporary studies, *Staphylococcus pseudintermedius* , *β‐hemolytic streptococci*, and *Pseudomonas aeruginosa* are consistently among the most frequently isolated pathogens. The highest MDR burdens are reported in referral populations and among methicillin‐resistant staphylococci worldwide. Feline data remain comparatively limited but show regional variability in resistance patterns, while equine studies reveal temporal shifts in isolate distribution and a rising prevalence of methicillin‐resistant organisms in tertiary settings. Recent topical antimicrobial exposure is the most consistently identified predictor of reduced culture positivity and elevated resistance rates in subsequent ocular isolates, highlighting the importance of early microbiologic sampling and judicious antibiotic use. Interpreting antimicrobial susceptibility testing (AST) in ophthalmology remains challenging because clinical breakpoints are generally derived from systemic dosing regimens, despite substantially higher, albeit transient, drug concentrations being achieved at the ocular surface following topical administration. Moreover, the ocular surface microenvironment, including tear proteins, inflammation, biofilm formation, and concurrent serum therapy, may substantially influence antimicrobial activity and therapeutic response. The review concludes with practical ophthalmology‐specific stewardship recommendations, a One Health perspective on resistant ocular pathogens, and a forward‐looking discussion of antibiotic‐sparing adjuncts within a broader multimodal strategy to preserve antimicrobial effectiveness.

## 1 Introduction

Antimicrobial resistance (AMR) has evolved from an abstract public health concern into a daily clinical challenge in veterinary ophthalmology. Bacterial ocular surface disease encompasses a spectrum from self‐limiting conjunctivitis to fulminant stromal infections characterized by enzymatic melting, descemetocele formation, perforation, and potential globe loss [1]. The cornea is uniquely vulnerable: it is avascular, continuously exposed to the environment, and reliant on tear film innate immunity, including lysozyme, lactoferrin, defensins, and immunoglobulins for antimicrobial defense [2]. Once epithelial integrity is compromised, bacterial proliferation within the anterior stroma can progress rapidly, particularly with protease‐producing and toxigenic organisms such as *Pseudomonas aeruginosa* and certain *β‐hemolytic streptococci* [1, 3, 4].

These biological dynamics create a familiar clinical tension: bacterial keratitis can threaten the globe and vision within hours to days, yet organism identification and antibiotic susceptibility test (AST) results typically require 48 to 72 h or longer. Consequently, empiric topical antimicrobial therapy is often initiated before culture and AST results are available [1, 5], a practice justified in cases of rapidly progressive disease where even a brief therapeutic delay may lead to irreversible damage. However, repeated or broad‐spectrum topical exposure exerts selective pressure at the ocular surface, potentially enriching resistant bacterial populations and contributing to the clinic‐level transmission of multidrug‐resistant (MDR) organisms, particularly in referral populations or patients with chronic ocular surface disease [4, 5, 6, 7]. Environmental sampling of ophthalmic clinics has demonstrated contamination of instruments and surfaces with MDR isolates, supporting the possibility of patient‐to‐patient transmission and hospital‐associated acquisition [8, 9].

Interpretation of AMR in ocular disease presents unique complexities. While AST in veterinary medicine follows standardized methodologies and increasingly incorporates pharmacokinetic/pharmacodynamic (PK/PD) principles for clinical interpretation [10, 11, 12, 13, 14], most ocular studies still use systemic breakpoints to classify a given isolate as susceptible or resistant. This is problematic because topical therapy can achieve ocular surface concentrations that, although transient and subject to rapid dilution and clearance, often exceed typical effective concentrations. Several factors further complicate the translation of in vitro susceptibility results into clinical response, including tear turnover, epithelial integrity, inflammation, protein binding, and biofilm‐associated tolerance [15, 16, 17, 18, 19]. The result is a recurring “topical breakpoint mismatch”, where isolates labeled resistant in vitro may respond to intensive topical dosing, while isolates appearing susceptible may fail if drug delivery or microenvironmental conditions reduce antimicrobial activity.

AMR in veterinary ophthalmology is therefore not merely a laboratory concept but a pharmacologically and epidemiologically nuanced clinical reality. It also provides a strong rationale for the ophthalmology community to engage seriously with antibiotic stewardship and antibiotic‐sparing alternatives, as traditional antimicrobial strategies are becoming progressively less reliable in clinical practice. In this context, this review focuses on bacterial diseases in dogs, cats, and horses, with an emphasis on bacterial keratitis. Our objectives are to: (i) summarize key principles of AST interpretation in topical ophthalmology; (ii) review molecular mechanisms of resistance relevant to ocular isolates; (iii) synthesize species‐specific pathogen ecology, susceptibility patterns, MDR prevalence, and temporal trends; (iv) consolidate evidence on risk factors for in vitro nonsusceptibility; and (v) provide practical stewardship and One Health considerations that set the stage for alternative approaches.

## 2 Antimicrobial Resistance and Susceptibility Testing in Ocular Disease

Antimicrobial resistance (AMR) is the ability of a microorganism to survive or proliferate despite exposure to an antimicrobial agent at concentrations that would normally inhibit or kill wild‐type strains [20]. This resistance is typically mediated by target‐site mutations, acquisition of resistance genes, efflux mechanisms, enzymatic degradation, altered permeability, or biofilm‐associated tolerance [20].

In veterinary ophthalmology, interpreting AMR requires a clear distinction between in vitro nonsusceptibility and clinical non‐response. In vitro nonsusceptibility is determined by AST using established breakpoints. Clinical non‐response, conversely, describes the failure of an infection to improve despite seemingly appropriate therapy. These two concepts do not always align. An isolate categorized as resistant based on systemic breakpoints may still respond to intensive topical dosing if tear film concentrations sufficiently exceed the minimum inhibitory concentration (MIC) for an adequate duration. Conversely, isolates categorized as susceptible may fail to respond due to inadequate stromal penetration, a high inoculum burden, biofilm formation, insufficient dosing frequency, or pharmacologic attenuation within the ocular microenvironment [18, 19].

### 2.1 Breakpoints and Topical Therapy

AST is the primary laboratory tool guiding targeted therapy for bacterial keratitis. While veterinary standards are methodologically harmonized (e.g., VetCAST and CLSI guidelines) and breakpoint frameworks increasingly incorporate PK/PD principles [10, 11, 12, 13, 14], these breakpoints are often derived from systemic dosing paradigms.

Topical ocular therapy results in a distinct pharmacokinetic profile. Immediately after instillation, tear film concentrations can be orders of magnitude higher than systemic peak serum levels [21, 22, 23]. However, these concentrations decline rapidly due to tear turnover, blinking, and nasolacrimal drainage. In dogs, these dynamics significantly reduce drug concentrations within minutes, especially in inflamed eyes [18, 22]. Thus, ocular pharmacodynamics are characterized by high, transient peaks followed by rapid clearance.

This discrepancy creates two interpretive risks. “False pessimism” occurs when systemic breakpoints classify an isolate as resistant despite clinical success with intensive topical dosing. “False optimism” arises when apparent in vitro susceptibility fails to translate into clinical efficacy due to limited stromal penetration, deep infection, biofilm‐associated tolerance, or inadequate dosing. Consequently, AST should inform therapeutic decisions but not dictate them.

### 2.2 Multidrug Resistance

Most veterinary ophthalmology studies define MDR using class‐based criteria aligned with Magiorakos et al. (2012), typically as resistance to at least one agent in three or more antimicrobial classes, while extensively drug‐resistant (XDR) isolates retain susceptibility to only one or two classes [24]. However, the prevalence of MDR is strongly influenced by study methods. Reported burden varies with the composition of AST panels, whether intermediate results are grouped as nonsusceptible, whether intrinsic resistance is counted, and which breakpoint standards are applied [25, 26, 27]. These factors limit direct comparisons across cohorts and institutions.

Despite these constraints, consistent trends emerge. MDR is most commonly documented in canine cohorts, particularly in referral settings. Methicillin‐resistant *Staphylococcus pseudintermedius* represents a disproportionate share of MDR isolates and frequently exhibits co‐resistance to macrolides, lincosamides, tetracyclines, and fluoroquinolones [6, 28, 29]. Resistance to older topical combinations, such as neomycin‐polymyxin B‐bacitracin and fusidic acid, is prevalent in contemporary datasets, diminishing their reliability as sole empiric therapy for stromal keratitis in many populations [4, 30].

### 2.3 Microenvironment of the Ocular Surface

Eyes with bacterial keratitis typically exhibit conjunctivitis and disruption of the blood‐tear barrier, allowing substantial leakage of serum albumin into the tear film compartment [16, 31, 32]. Albumin concentrations in diseased tears can significantly reduce the free (microbiologically active) fraction of topically administered drugs, thereby limiting ocular penetration and bioavailability [18, 33]. In vitro, albumin increases MICs in a dose‐, pathogen‐, and antibiotic‐dependent manner [18], and similar modulation is observed when topical blood‐derived products are introduced into susceptibility systems [34]. Collectively, these findings provide a mechanistic explanation for clinical–laboratory discordance and help elucidate why high tear concentrations do not necessarily translate into effective in vivo antimicrobial activity. For example, an isolate may be classified as susceptible to ofloxacin based on standard AST, yet the corresponding clinical case may continue to deteriorate despite frequent topical administration. This phenomenon has been demonstrated in a recent report of canine bacterial keratitis, where 93% of isolates were susceptible to at least one administered antibiotic, yet unfavorable clinical outcomes still occurred, including progression of corneal disease, need for surgical stabilization, and even globe loss [18].

Bacterial organization within the infected cornea may further affect antimicrobial activity and clinical outcomes. Biofilm formation is increasingly recognized among ocular isolates, particularly *staphylococci* and *Pseudomonas aeruginosa* , and is associated with reduced antimicrobial susceptibility despite favorable AST results [19, 35]. This helps explain clinical failure in chronic or refractory ulcers.

### 2.4 Practical Interpretation of AST and MDR

Interpretation of AST results in ocular infections should extend beyond simple susceptible/resistant categorization and incorporate organism identity, MIC values (when available), ulcer depth and severity, cytological findings, prior antimicrobial exposure, and the early clinical response during the first 24–48 h of treatment [1, 4, 28, 36]. This integrated approach better reflects real‐world ocular pharmacology and clinical decision‐making. To maximize the clinical value of laboratory testing, submissions should clearly indicate that the sample originates from a corneal ulcer or infectious keratitis and include relevant clinical details such as ulcer depth, presence of stromal melting, prior antimicrobial therapy, and suspected disease progression. Laboratories should be encouraged to provide species‐level identification, MIC values, and susceptibility testing for antimicrobials commonly used topically in the region. Early communication of preliminary identification results can facilitate timely refinement of therapy before final AST reporting is available.

In this context, MDR should be viewed as a marker of increased clinical burden rather than a reliable predictor of therapeutic failure. In canine keratitis caused by *Staphylococcus pseudintermedius* , MDR status was associated with longer follow‐up but was not linked to poorer globe or vision preservation, greater ulcer severity, or an increased need for surgical intervention [28]. In human corneal infections, higher MICs have been associated with delayed epithelial healing and more pronounced corneal scarring [37, 38]. Thus, MDR does not uniformly translate into treatment failure; rather, it may reflect a more prolonged disease course and, in some cases, an increased risk of clinically meaningful sequelae such as corneal scarring.

## 3 Mechanisms of Resistance in Ocular Isolates

Resistance determinants in ocular isolates are frequently carried on mobile genetic elements (e.g., plasmids, transposons, and integrative chromosomal elements), facilitating horizontal gene transfer and the spread of multidrug resistance [39, 40]. Mechanistically, ocular antimicrobial resistance arises through target‐site mutations, acquisition of resistance genes, enzymatic drug inactivation, altered permeability and efflux, and biofilm‐mediated tolerance. Although keratitis management remains organism‐directed rather than gene‐directed, understanding these mechanisms helps contextualize susceptibility patterns, explain inter‐center variability, and reconcile laboratory findings with clinical outcomes.

### 3.1 Staphylococci

Methicillin resistance in *Staphylococcus pseudintermedius* (MRSP) and *Staphylococcus aureus* (MRSA) is primarily mediated by the *mecA* gene (less commonly *mecC*), which encodes the altered penicillin‐binding protein PBP2a with reduced β‐lactam affinity [6, 28, 29]. In contrast, resistance to penicillin and other narrow‐spectrum β‐lactams is typically mediated by *blaZ*, which encodes a β‐lactamase capable of hydrolyzing the antibiotic prior to target binding; these determinants may occur independently or co‐segregate within the same isolate [29]. In ophthalmic isolates, *mecA* positivity is strongly associated with multidrug resistance, frequently encompassing macrolides, lincosamides, tetracyclines, aminoglycosides, and fluoroquinolones [28, 29, 41, 42].

Beyond *mecA* and *blaZ*, whole‐genome sequencing studies of ocular *S. aureus* have identified a diverse resistome that includes *erm* methyltransferases, *msr(A)/mphC*, aminoglycoside‐modifying enzymes, *tet(K/M)*, *fusB/C*, *dfr* genes, and multiple efflux regulators. In contrast, comparable genomic characterization of veterinary ocular isolates remains limited [43]. Fluoroquinolone resistance typically arises through point mutations within the quinolone resistance‐determining regions (QRDRs) of *gyrA* and *parC*, sometimes compounded by efflux activity (e.g., NorA), resulting in incremental increases in MICs under repeated topical exposure [36, 42].

Biofilm formation provides an additional layer of antimicrobial tolerance. Biofilm‐associated staphylococci demonstrate reduced antimicrobial penetration and altered metabolic activity, contributing to persistence despite favorable AST results [19]. Clonal lineages including ST71 and emerging types such as ST258 and ST496 are increasingly associated with complex MDR phenotypes [28, 42].

### 3.2 Streptococci

*β‐hemolytic streptococci* are important ocular pathogens in domestic species. Resistance mechanisms in *β‐hemolytic streptococci* include stepwise mutations in penicillin‐binding protein genes (e.g., *pbp2x*, *pbp2b*) leading to reduced β‐lactam susceptibility, as well as acquisition of mobile macrolide resistance determinants such as *erm(B)*, *erm(TR)*, and *mef(A)*. Tetracycline resistance genes (*tetM*, *tetO*) frequently co‐localize on Tn916‐like integrative conjugative elements, facilitating horizontal dissemination [44]. Strain‐level variation appears clinically relevant: *Streptococcus canis* sequence type 43 (ST43) has been associated with progressive collagenolysis and conjunctival graft failure despite aggressive therapy, potentially due to a fructose phosphotransferase system that enhances metabolic fitness within the corneal microenvironment [45].

### 3.3 Gram‐Negative Organisms

*Pseudomonas aeruginosa* is a clinically important ocular pathogen because of its virulence and complex resistance mechanisms [46]. Its intrinsic resistance includes low outer‐membrane permeability, production of chromosomal *AmpC* β‐lactamase, and multidrug efflux pumps such as *MexAB‐OprM* [46]. Overexpression of *MexAB‐OprM* commonly results from regulatory mutations in *mexR*, *nalB*, *nalC*, or *nalD*, while the loss or mutation of the OprD porin confers resistance to carbapenems like imipenem [46]. Fluoroquinolone resistance typically arises through mutations in DNA gyrase and topoisomerase IV (e.g., *gyrA* and *parC*), including commonly reported quinolone resistance‐determining regions (QRDR) mutations such as gyrA Thr83‐Ile in canine isolates [47]. As in staphylococcal infections, biofilm formation is common in *Pseudomonas aeruginosa* keratitis [48], with structured communities embedded in an extracellular matrix that restricts antimicrobial penetration, alters metabolic activity, and promotes tolerance beyond that predicted by AST results.

Enterobacterales in the eye may also harbor extended‐spectrum β‐lactamases (ESBLs) [49, 50]. Although their reported prevalence in ocular isolates is lower than in systemic infections, ESBL‐producing *Escherichia coli* raises concerns due to their zoonotic potential and resistance to extended‐spectrum cephalosporins [49, 50, 51]. Genomic similarities between animal and human isolates further reinforce the One Health importance of monitoring these pathogens in the ocular environment [52, 53].

## 4 Epidemiology and Susceptibility Patterns

### 4.1 Dogs

In contemporary canine keratitis cohorts, a consistent triad of pathogens predominates: *Staphylococcus pseudintermedius* , β‐hemolytic streptococci (particularly *Streptococcus canis* ), and *Pseudomonas aeruginosa* [4, 5, 7, 30, 54, 55, 56, 57]. While the relative frequency of these organisms appears broadly stable across regions [5], the resistance landscape within this spectrum is increasingly dynamic and has become a defining feature of referral canine keratitis populations [4, 6, 55]. When stratified by continent, MDR prevalence demonstrates substantial regional heterogeneity but consistently reaches clinically meaningful levels, particularly in tertiary and referral settings that are enriched for progressive or previously treated ulcers.

Across North American referral populations, the dominant triad of pathogens in canine ulcerative keratitis consists of *Staphylococcus pseudintermedius* , *β‐hemolytic streptococci*, and *Pseudomonas aeruginosa* . Earlier data from the Southeastern US (1993–2003; 97 dogs) showed a similar pathogen distribution, with no significant temporal increase in antimicrobial resistance over that decade [57]. However, more recent data demonstrate a different trajectory for resistance. In a referral population from the northeastern United States (New York), 23.9% of corneal *Staphylococcus* isolates from dogs with keratitis were methicillin‐resistant and exhibited extensive multidrug resistance, particularly to fluoroquinolones [6]. In a 2014–2020 cohort (476 dogs), MDR was identified in 20% of isolates overall, increasing from 5% to 34% over 5 years, despite a stable distribution of organisms [4]. A subsequent Iowa referral cohort (2018–2021; 187 dogs) similarly reported 20% MDR, with an additional 18% of isolates possibly being extensively drug‐resistant; although MDR proportions remained stable (19% vs. 20%), acquired resistance increased significantly over time [5]. MIC‐based analysis from Ohio (2013–2018; 134 dogs) further demonstrated significant increases in median MIC values over time for erythromycin and trimethoprim‐sulfa among *Staphylococcus* spp., and for moxifloxacin among *Pseudomonas* spp. [36]. Prior or current topical fluoroquinolone exposure was associated with significantly higher MICs across multiple fluoroquinolones in canine *Staphylococcus* isolates, indicating measurable selection pressure [36]. Collectively, North American data suggest a transition from relative resistance stability in earlier decades to contemporary MDR rates that are consistently reported in referral cohorts, with documented escalation within individual centers and clear evidence of progressive susceptibility erosion, particularly among staphylococci following fluoroquinolone exposure.

South American data, largely from Brazil, demonstrate a pathogen spectrum in canine ulcerative keratitis similar to other regions, with *Staphylococcus* spp. (particularly *S. pseudintermedius* ), *β‐hemolytic streptococci*, and *Pseudomonas aeruginosa* predominating. In São Paulo State, MDR was identified in 23.3% of isolates from dogs with ulcerative keratitis, with staphylococci representing the most frequently cultured genus [56]. In Rio de Janeiro, 42.5% of staphylococcal isolates from external ocular infections met MDR criteria, and 61.1% of *S. intermedius* strains were oxacillin‐resistant, consistent with methicillin resistance [58]. Similarly, in Paraná, 69.2% of ophthalmic isolates demonstrated multidrug resistance, and among staphylococci, 61.1% were phenotypically methicillin‐resistant, with 38.9% harboring the *mecA* gene [41]. Together, Brazilian studies reveal marked intra‐country heterogeneity, with moderate MDR prevalence in recent keratitis cohorts but substantially higher multidrug and methicillin resistance among staphylococci when broader external ocular diseases are analyzed.

In Europe, the escalation of resistance has been particularly striking. In Switzerland (2009–2013; 89 dogs), *Staphylococcus* spp. and *Streptococcus* spp. accounted for two‐thirds of isolates in septic keratitis, and nearly 50% of canine Staphylococcus isolates were resistant to second‐generation fluoroquinolones [59]. In the Netherlands (122 dogs), MDR increased from 9.4% (2012–2015) to 38.6% (2016–2019) despite a stable distribution of organisms [55], reinforcing that resistance patterns cannot be inferred solely from pathogen identity. UK referral cohorts similarly report *Staphylococcus pseudintermedius* , *Streptococcus canis* , and *Pseudomonas aeruginosa* as the dominant pathogens in progressive canine keratitis, with *P. aeruginosa* associated with malacic ulcers and globe loss [30, 35, 60]. In vitro resistance to neomycin and fusidic acid was common, while gentamicin and fluoroquinolones maintained relatively favorable susceptibility profiles [30, 35].

Asian studies show significant variations, with some centers reporting very high resistance levels. In South Korea, Kang et al. (2014) detected the *blaZ* gene in 92% and the *mecA* gene in 36% of ophthalmic *Staphylococcus pseudintermedius* isolates, reflecting a substantial burden of β‐lactam resistance gene carriage [29]. A more recent keratitis cohort from South Korea reported that 52.5% of isolates met MDR criteria [7]. In Thailand, bacteria were isolated from 81.3% of severe ulcers, and 90.9% of staphylococcal isolates carried *mecA*, indicating methicillin resistance; more than 50% were resistant to all tested fluoroquinolones, and ESBL genes were detected in Gram‐negative isolates [50]. Data from Taiwan (190 ulcerated eyes) showed a 71% culture positivity rate, with *Staphylococcus* spp. accounting for 49% of isolates and frequent resistance to commonly used ophthalmic agents, although ciprofloxacin retained activity against most organisms except streptococci [61]. Other regional reports include high quinolone resistance in India (88.9%) and significant tetracycline resistance (96.6%) with frequent biofilm production among *Staphylococcus pseudintermedius* isolates in China [19]. Additional data from China on canine bacterial conjunctivitis also identified frequent carriage of aminoglycoside resistance genes (e.g., *aacA‐aphD*), even though some aminoglycosides retained in vitro susceptibility [62]. Overall, Asian cohorts report some of the highest documented rates of MDR and methicillin‐resistant staphylococci, with particularly pronounced fluoroquinolone resistance in specific regions.

Collectively, these continent‐specific data highlight the dynamic, region‐ and center‐dependent nature of antimicrobial resistance in canine keratitis, with multiple studies documenting measurable increases over relatively short timeframes. Ulcer phenotype and seasonality further influence the epidemiology and resistance burden. Progressive or malacic ulcers are disproportionately associated with *Pseudomonas aeruginosa* , reflecting its protease‐mediated virulence and capacity for rapid stromal destruction [30, 35]. Greater ulcer depth and surface area have also been associated with the isolation of antimicrobial‐resistant bacteria, supporting a link between lesion severity and resistance phenotype [50]. Seasonal increases in *Pseudomonas* sp. isolation have also been reported during warmer months [4, 30].

Taken together, contemporary canine data support three central principles. First, MDR is no longer uncommon in referral keratitis populations across multiple continents, with prevalence typically around 20%–25% but reaching 40%–50% in selected referral settings. Second, resistance cannot be reliably predicted based solely on organism identity, particularly within *staphylococci*. Third, local and regularly updated surveillance is essential for rational empiric therapy, as both “acquired resistance” and MDR proportions can evolve rapidly over time and vary by ulcer phenotype and region.

### 4.2 Cats

Compared with dogs, feline‐specific surveillance data for bacterial keratitis remain limited, with most studies being small and heterogeneous in design. The largest feline keratitis cohort to date is a North American referral study of 81 cats (2004–2017) with 102 aerobic corneal isolates, where *Staphylococcus* spp. predominated and in vitro susceptibility remained high for commonly used topical agents [63]. European data provide additional context. In a Netherlands referral cohort (2012–2019; 33 cats), isolates were primarily *Staphylococcus* spp. (largely *S. felis* ), with fewer β‐hemolytic streptococci and rare *Pseudomonas aeruginosa* . Only one isolate (3%) met the study's MDR definition, and no methicillin‐resistant staphylococci were detected [55]. In Switzerland (2009–2013; 28 cats), isolates from feline septic keratitis similarly consisted mainly of *Staphylococcus* spp. and *Streptococcus* spp. [59]. Methicillin resistance was suspected in two isolates, with one confirmed as MRSP, indicating that resistant staphylococci can occur despite overall favorable susceptibility patterns [59]. Asian data further illustrate geographic variability. In Taiwan (2000–2006), 92 cats with infected ulcerative keratitis yielded 59 isolates, with Gram‐positive isolates outnumbering Gram‐negative isolates by approximately 3:1, although MDR prevalence was not explicitly reported [64].

Interpretation of feline susceptibility data requires additional caution because feline ocular surface disease is often associated with feline herpesvirus‐1 (FHV‐1), where bacterial growth may represent secondary colonization rather than primary invasive infection [65]. Overall, current evidence suggests that feline keratitis isolates often retain favorable susceptibility profiles, particularly when compared to canine cohorts. However, limited case numbers, heterogeneous methodologies, and infrequent MDR‐focused analyses preclude firm conclusions regarding the global feline MDR burden.

### 4.3 Horses

Equine infectious keratitis presents a distinct epidemiologic landscape shaped by environmental exposure, climate, and the frequent coexistence of bacterial and fungal pathogens. Across referral cohorts, Gram‐positive organisms predominate, particularly *Staphylococcus* spp. and *Streptococcus* spp., most commonly *Streptococcus equi* subsp. *zooepidemicus*, with variable representation of Gram‐negative rods including *Pseudomonas* spp. [66, 67, 68, 69, 70, 71].

Clinically meaningful resistance is consistently reported across regions, although patterns vary considerably between centers. Earlier North American data from Tennessee (1993–2004; 43 horses) identified *Streptococcus equi subsp. zooepidemicus* and *Pseudomonas aeruginosa* as leading pathogens, with preserved fluoroquinolone and aminoglycoside susceptibility, but reduced bacitracin activity in *streptococci*, a pattern associated with frequent prior exposure to neomycin‐polymyxin B‐bacitracin therapy [66].

In the Netherlands (2012–2021; 178 horses), *Staphylococcus* spp. and *Streptococcus* spp. predominated in referral ulcerative keratitis, with multidrug resistance observed in 26% of bacterial isolates; although acquired resistance was relatively common, the overall incidence of resistance did not significantly increase over the nine‐year study period [71]. In Belgium (2014–2021; 196 horses), *Staphylococcus* spp. and *Streptococcus* spp. also predominated, with approximately half of *S. aureus* isolates being methicillin‐resistant [67]. Overall resistance was low to chloramphenicol and fluoroquinolones, but substantially higher to several commonly used topical agents. Methicillin‐resistant isolates demonstrated broader multidrug resistance patterns, emphasizing the implications for infection control and antibiotic stewardship [67]. In Finland (2007–2018; 20 eyes), infectious keratitis cases were dominated by *Streptococcus equi subsp. zooepidemicus*, and all tested bacterial isolates were susceptible to penicillin G and chloramphenicol [68]. *Streptococci* exhibited predictable resistance to fusidic acid and variable susceptibility to tetracycline [68]. Longitudinal surveillance from the United Kingdom (2012–2019; 110 horses) showed that chloramphenicol resistance increased from 0% to 30%, while ofloxacin resistance decreased across study periods, highlighting the influence of local prescribing patterns and selective pressures [69].

In Australia (2010–2020; 38 horses), *Pseudomonas* spp. represented a substantial proportion of isolates in referral ulcerative keratitis, with amikacin retaining strong in vitro activity, whereas older combination products containing polymyxin B or neomycin demonstrated reduced in vitro coverage against some isolates [70].

Methicillin‐resistant staphylococci are increasingly documented in equine referral settings and may exhibit resistance across multiple drug classes, paralleling MRSA/MRSP concerns in small animal practice [59, 67]. Because equine ulcers are often managed by multiple practitioners before referral and may require prolonged therapy, cumulative antimicrobial exposure and referral bias must be considered when interpreting resistance prevalence [69].

Equine keratitis fundamentally differs from small animal disease due to the frequent presence of fungal or mixed infections, necessitating early cytology and culture (including fungal culture where indicated) to avoid prolonged “antibacterial failure” that is actually driven by fungal disease [68, 71, 72].

Taken together, equine data support three main concepts: first, regional ecology and prescribing practices strongly shape antimicrobial susceptibility patterns. Second, methicillin‐resistant staphylococci appear to be clinically relevant in equine keratitis referral populations. Third, because mixed infections and prior antimicrobial exposure are common, early culture (bacterial and fungal where indicated) is essential for stewardship and for avoiding prolonged empiric therapy unsupported by local data.

## 5 Risk Factors for In Vitro Nonsusceptibility and MDR

Understanding the risk factors for nonsusceptibility is clinically important because it informs when culture and AST are essential, how empiric therapy should be structured, and which cases warrant early reassessment or escalation.

### 5.1 Prior Topical Antimicrobial Exposure

Recent topical antimicrobial exposure is the most consistently documented risk factor for in vitro nonsusceptibility, operating through class‐selective pressure. For instance, prior fluoroquinolone use has been associated with significantly increased MIC values in subsequent canine ocular isolates [36]. Similarly, prior chloramphenicol therapy was significantly associated with acquired chloramphenicol resistance in dogs with stromal ulcerations, illustrating selection under sustained ocular surface exposure [55]. Prospective data further support this dynamic: dogs treated with topical ofloxacin for 3 weeks after cataract surgery exhibited a shift in conjunctival flora toward coagulase‐negative staphylococci, with a transient but significant increase in fluoroquinolone‐resistant isolates during therapy [73]. Comparable patterns have been observed in equine ulcerative keratitis, where decreased susceptibility to bacitracin and gentamicin was noted in horses previously exposed to triple‐antibiotic or gentamicin formulations, respectively [66, 71]. Beyond resistance selection, veterinary studies demonstrate that topical antibiotic therapy can induce measurable shifts in ocular surface microbial community structure, with a partial or complete return to baseline after drug discontinuation [74, 75, 76, 77]. Recent therapy also reduces culture positivity and may distort the spectrum of recovered pathogens [30, 55], introducing an interpretive bias in surveillance datasets where the isolates recovered are likely the most resistant survivors.

### 5.2 Procedural and Hospital Exposures

Procedural and environmental exposures represent a second risk domain. In a canine cohort focused on *Staphylococcus pseudintermedius* keratitis, MDR was significantly associated with general anesthesia within the preceding 30 days; dogs with MDR were significantly more likely to have undergone recent anesthesia than those with non‐MDR infections [28]. Although causality could not be established in that retrospective study, plausible mechanisms included perioperative antimicrobial exposure, reduced tear production during and after anesthesia, and exposure to shared equipment and environmental reservoirs (i.e., nosocomial transmission). Environmental reservoirs within ophthalmic clinics further support the possibility of healthcare‐associated acquisition. Contamination of ophthalmic equipment and clinic surfaces with MDR staphylococci has been documented, with evidence of shared or genetically similar strains between environmental and patient isolates [8, 9].

### 5.3 Host and Ocular Surface Factors

Host and ocular surface factors influence both pathogen risk and resistance dynamics. Chronic ocular surface disease predisposes patients to repeated antimicrobial exposure and altered local immunity. Keratoconjunctivitis sicca (“dry eye”) not only reduces tear volume but also diminishes protective antimicrobial proteins, such as lysozyme and lactoferrin, thereby weakening innate defenses [78, 79]. Brachycephalic conformation increases the risk of exposure keratopathy and recurrent ulceration [59, 80, 81], amplifying cumulative antibiotic exposure over time. In addition to intrinsic factors, household and occupational exposures may also shape resistance risk. Dogs owned by individuals employed in veterinary or human healthcare fields are 4.6 times more likely to present with methicillin‐resistant *Staphylococcus* keratitis compared to those owned by individuals in other professions, suggesting potential bidirectional zoonotic transmission and shared environmental reservoirs [6].

## 6 Diagnostic Strategy and Therapeutic Intervention

Effective management of bacterial keratitis requires the integration of timely sampling, contextual interpretation of microbiologic data, and severity‐stratified empiric therapy.

### 6.1 Sampling Strategy

Microbiologic sampling is valuable in clinical practice [1, 60, 82, 83]. Corneal cultures obtained before extensive topical antibiotic exposure maximize yield and reduce interpretive ambiguity [82]. In referral populations, prior therapy is common and reduces culture positivity, potentially enriching for organisms that persist despite treatment [30, 55]. Therefore, antimicrobial history, dosing frequency, and timing of the most recent instillation should be documented and incorporated into AST interpretation. Cytology remains clinically useful when interpreted by experts. Identifying bacteria, particularly intracellular organisms associated with neutrophilic inflammation, supports active infection and can help distinguish cocci from rods while culture results are pending. However, sensitivity is moderate, and false‐negative results can occur; thus, cytology and culture should be viewed as complementary [60]. Corneal sampling should target the ulcer bed and advancing stromal margin to minimize the recovery of colonizing flora.

### 6.2 Ocular Antibiotics

Effective antibiotic therapy at the ocular surface requires consideration not only of bacterial susceptibility but also of antibiotic pharmacologic properties (Table 1). Clinicians should understand the primary activity of the selected antimicrobial(s). Bacteriostatic antibiotics may theoretically antagonize bactericidal agents by inhibiting bacterial growth and thereby reducing the activity of drugs that depend on active cell division [84]. Accordingly, avoiding bacteriostatic‐bactericidal combinations may be prudent; however, the clinical relevance of this interaction in topical ophthalmic therapy remains uncertain. Furthermore, antibiotics used in veterinary ophthalmology differ in their mechanisms of action and pharmacokinetic/pharmacodynamic (PK/PD) drivers, which may be broadly classified as time‐dependent (T>MIC), concentration‐dependent (Cmax/MIC), or exposure‐dependent (AUC/MIC) [14, 85]. These distinctions are particularly relevant in topical therapy, where drug concentrations are high but short‐lived due to rapid precorneal clearance [21, 22, 23, 86, 87, 88, 89].

**TABLE 1:** Topical antibiotics in veterinary ophthalmology: Mechanisms of action, antibacterial spectrum, pharmacodynamic behavior, and practical considerations.

Antibiotic class	Commonly used drugs	Activity	Mechanisms of action	Main antibacterial spectrum	PK/PD pattern	Dominant PK/PD index	Practical considerations
Aminoglycosides	Amikacin Gentamicin Neomycin Tobramycin	Bactericidal	Binds 30S ribosomal subunit → mistranslation, inhibition of protein synthesis	Broad spectrum; *Staphylococci*, Gram‐negative rods (*Pseudomonas*, *Proteus*)	Concentration‐dependent	Cmax/MIC	Notable post‐antibiotic effect. Gentamicin can be epitheliotoxic with frequent use. Synergy with cephalosporins.
Fluoroquinolones	Ciprofloxacin Gatifloxacin Moxifloxacin Ofloxacin	Bactericidal	Inhibits DNA gyrase and topoisomerase IV	Broad spectrum; second gen (cipro/oflo) stronger Gram‐negative/*Pseudomonas* activity; 4th gen (gati/moxi) better for Gram‐positive	Exposure‐dependent	AUC/MIC (secondary Cmax/MIC)	Notable post‐antibiotic effect. Good corneal penetration. Neutral/synergistic with other bactericidals.
Cephalosporins	Cefazolin Ceftazidime	Bactericidal	Inhibits cell wall synthesis (penicillin‐binding proteins)	Cefazolin: Gram‐positive; Ceftazidime: *Pseudomonas*	Time‐dependent	Time>MIC	Requires compounding (fortified). Synergy with aminoglycosides.
Phenicols	Chloramphenicol	Bacteriostatic (usually)^a^	Binds 50S → inhibits peptidyl transferase	Broad spectrum, including anaerobes and intracellular pathogens. Limited activity against *Pseudomonas*	Time‐dependent	Time>MIC (secondary AUC/MIC)	Good corneal penetration. Careful handling (rare risk of aplastic anemia in humans).
Tetracyclines	Chlortetracycline Doxycycline Oxytetracycline Tetracycline	Bacteriostatic	Binds 30S → inhibits protein synthesis	Broad spectrum; good for intracellular bacteria (*Mycoplasma* spp., *Chlamydia* spp.)	Exposure‐dependent	AUC/MIC	Anti‐collagenolytic effects at high concentrations.
Polypeptides	Bacitracin Gramicidin Polymyxin B	Bactericidal	Membrane disruption (polymyxin/gramicidin); cell wall inhibition (bacitracin)	Bacitracin/gramicidin: Gram‐positive cocci Polymyxin: Gram‐negative/*Pseudomonas*	Concentration‐dependent	Cmax/MIC	Polymyxin B: rare anaphylaxis in cats. Bacitracin only ointment; gramicidin solution.
Fusidanes	Fusidic acid	Bacteriostatic (usually)^a^	Inhibits elongation factor G	Narrow spectrum; Gram‐positive cocci (*Staphylococcus* sp.)	Time‐dependent	Time>MIC	Good corneal penetration. Synergy with aminoglycosides and macrolides.
Sulfonamides/Anti‐folate agents	Sulfacetamide Trimethoprim	Bacteriostatic	Inhibits folic acid synthesis	Broad spectrum when combined; limited activity against *Pseudomonas*	Time‐dependent	Time>MIC	Sulfacetamide may irritate and delay healing. Synergy with combination.
Macrolides	Azithromycin Erythromycin	Bacteriostatic	Binds 50S → inhibits protein synthesis	Mainly Gram‐positive; good for intracellular bacteria (*Mycoplasma* sp., *Chlamydia* sp.)	Exposure‐dependent	AUC/MIC	Notable post‐antibiotic effect. Azithromycin long tissue half‐life; erythromycin common for feline conjunctivitis.
Glycopeptides	Vancomycin	Bactericidal	Binds D‐Ala‐D‐Ala → inhibits cell wall synthesis	Narrow spectrum; Gram‐positive only	Time‐dependent	Time>MIC	Last‐resort antibiotic. Restricted usage under stewardship guidelines.

*Note:* Antibiotic activity, dominant PK/PD indices, and synergy data are largely derived from systemic or in vitro antimicrobial susceptibility studies. Their relevance to topical ophthalmic therapy should be interpreted cautiously, as ocular surface exposure is characterized by high transient peak concentrations and short residence times, and is strongly influenced by tear turnover, blinking, nasolacrimal drainage, epithelial integrity, and the local microenvironment.

Abbreviations: AUC, area under the curve; Cmax, maximal concentration; MIC, minimal inhibitory concentration; PK/PD, pharmacokinetic/pharmacodynamic.

^a^ Can exhibit bactericidal activity against certain pathogens at high concentrations.

### 6.3 Ocular Antibiograms

Hospital‐wide antibiograms predominantly reflect systemic infections and may not represent ocular isolates [90]. Ocular‐specific antibiograms more accurately capture corneal pathogen distribution, cumulative antimicrobial exposure, and referral bias [4, 69]. Ideally, they should be specimen‐stratified (corneal vs. conjunctival) and updated regularly. Referral centers, enriched with severe or previously treated ulcers, may report higher proportions of MDR organisms than first‐opinion practices. Surveillance data should therefore be contextualized rather than generalized across practice settings. A recent viewpoint further emphasizes that aggregated, ophthalmology‐specific antibiograms can make local resistance patterns visible to clinicians and represent a practical, immediately actionable tool to support antimicrobial stewardship in veterinary ophthalmology [91].

### 6.4 Severity‐Stratified Empiric Therapy

Empiric therapy should reflect the severity of the ulcer, its phenotype, and local epidemiology. Superficial ulcers may be managed with targeted topical therapy, with culture reserved for cases that fail to improve within 24–48 h. In contrast, stromal ulcers exhibiting marked infiltration, keratomalacia, or rapid progression necessitate early culture and broad empiric coverage, including activity against Gram‐positive cocci and Gram‐negative rods [4, 5]. This is broadly consistent with human ophthalmology guidance, where small bacterial keratitis cases are commonly managed empirically, whereas culture is recommended for large, central, atypical, or poorly responsive ulcers [90]. Early clinical response is crucial; improvement supports de‐escalation once culture results are available, while a lack of response should prompt reassessment of coverage, penetration, dosing frequency, biofilm involvement, or the need for surgical intervention. Intensive early dosing helps maintain drug concentrations above MIC during the highest‐risk phase. As the infection stabilizes, dosing intervals may be extended to reduce epithelial toxicity and minimize antimicrobial selective pressure.

### 6.5 Systemic Antibiotics and the Ocular Surface

A persistent misconception in both general practice and referral settings is that systemic antibiotics provide therapeutically meaningful concentrations in the tear film for managing corneal infections. Systemic drugs can enter the tear compartment, but the magnitude and clinical relevance of this exposure are often limited and strongly influenced by the integrity of the blood‐tear barrier.

Evidence from veterinary studies illustrates this problem. Following oral administration of doxycycline in dogs, the drug can be detected in the tears but levels are generally low compared to what would be expected for direct antimicrobial activity in keratitis [92, 93]. This is consistent with pharmacokinetic data in cats, where orally administered doxycycline achieved minimal or undetectable tear concentrations despite adequate systemic exposure, whereas pradofloxacin reached higher tear concentrations [94]. In another report, ceftiofur was detectable in dog tears for up to 10 days after subcutaneous administration of ceftiofur crystalline‐free acid, yet tear concentrations remained below MICs for common ocular isolates in that study [95].

This does not preclude a role for systemic antibiotics in selected cases. They may be appropriate when infection involves vascularized corneal defects, corneal perforation with the risk of intraocular extension, periocular or orbital tissues, or following corneal surgeries such as conjunctival pedicle flap placement, where drug delivery via systemic circulation targets tissues beyond the tear film alone.

## 7 One Health Considerations and Infection Control

Resistant ocular pathogens must be considered within a One Health framework, as antimicrobial exposure, resistant organisms, and resistance determinants circulate across human, animal, and environmental interfaces [8, 9, 53, 96, 97]. Antimicrobial use in veterinary ophthalmology thus contributes to a shared ecological system in which high‐risk clones and mobile resistance genes may disseminate beyond the individual patient.

This is particularly relevant for *Staphylococcus pseudintermedius* , the most common canine ocular isolate. Once regarded as largely canine‐adapted, it is now recognized as a zoonotic and frequently underdiagnosed human pathogen [98, 99, 100, 101]. Human infections have been documented, especially among dog owners and veterinary personnel, and multidrug‐resistant strains, including MRSP, are increasingly reported [98, 99, 100, 101, 102]. Genomic and epidemiologic data further indicate that dominant clonal lineages are not strictly host‐restricted, supporting the potential for interspecies transmission [101].

Veterinary clinics and households may function as interconnected transmission networks. Resistant organisms can persist on hands, examination tables, tonometer tips, diagnostic lenses, drop bottles, and other ophthalmic instruments if disinfection protocols are inadequate [8, 9]. Close contact during restraint, topical drug administration, and postoperative care increases opportunities for bidirectional exchange between animals and humans. In this context, ocular AMR represents not only a therapeutic challenge but also a significant infection control concern.

Effective infection control requires validated disinfection protocols with defined contact times, rigorous hand hygiene, and systematic decontamination of all equipment that contacts the ocular surface [8, 9, 97, 103, 104]. Using dedicated instrument sets for infected cases, employing single‐use tonometer covers when appropriate [9], and implementing workflow separation for high‐risk ulcers may reduce cross‐transmission [9]. Environmental surveillance should be considered when clusters of multidrug‐resistant keratitis are suspected [8].

One Health and infection control serve complementary purposes: protecting individual patients within the clinic while limiting the dissemination of resistant organisms across the animal‐human interface [6, 8, 53, 103].

## 8 Antibiotic Stewardship in Veterinary Ophthalmology

As antimicrobial resistance rises, stewardship must be regarded as a core clinical competency rather than an abstract principle. In ophthalmology, where intensive topical therapy, fast disease dynamics, and close human‐animal contact coexist, even small steps can have a significant clinical and public health impact. The following principles translate stewardship into practical daily decision‐making:

- **Culture early** in any stromal ulcer where infection is plausible, especially before changing or “stacking” antibiotics. This includes ulcers with stromal infiltrate, malacia, rapid progression, deep defects, prior treatment failure, or high‐risk histories (e.g., recent topical antimicrobials, recent anesthesia, chronic ocular surface disease, owner in healthcare profession).
- **Document antibiotic exposure precisely**. Agent(s), frequency, duration, and time since last instillation should be recorded, as recent topical therapy reduces culture yield and may bias the recovered pathogen spectrum.
- **Interpret AST in clinical context**. Do not allow a binary susceptible/resistant label to override clinical findings and pharmacologic realities. When available, integrate MIC values, ulcer phenotype, and early clinical trajectory while being aware of topical “breakpoint mismatch”, biofilm‐associated tolerance, and tear protein effects.
- **Escalate promptly when severity warrants but de‐escalate once safe**. Once clinical stabilization occurs and culture results return, narrow therapy to reduce toxicity and selection pressure.
- **Avoid routine broad‐spectrum prophylaxis** in noninfectious conditions. When prophylaxis is necessary, select the narrowest effective approach and establish a clear endpoint.
- **Reserve systemic therapy for rare, clearly justified circumstances** rather than using it as routine coverage. Systemic antibiotics are generally unnecessary for corneal infections in domestic species, as tear concentrations often remain below MICs for common keratitis pathogens.
- **Embed infection‐control behavior into the ophthalmology workflow**. Rigorous hand hygiene, validated and frequent equipment disinfection, and separation of high‐risk infected cases all help reduce patient‐to‐patient transmission and lower downstream antimicrobial demand.
- **Integrate antibiotic‐sparing adjuncts when appropriate**. The challenge lies not only in the emergence of resistant organisms but also in the diminishing reliability of empiric antibiotic strategies. In cases of refractory, chronic, recurrent, melting, or MDR‐associated keratitis, antibiotic‐sparing adjuncts may help reduce microbial burden, enhance antimicrobial activity, disrupt biofilms, or limit cumulative antibiotic exposure. These include topical antiseptics and antimicrobial‐potentiating formulations such as povidone‐iodine, hypochlorous acid, polyhexanide, hexamidine, ozone, and EDTA‐based combinations (e.g., Tris‐EDTA formulations), as well as emerging non‐pharmacologic modalities including PACK‐CXL, cold atmospheric plasma, and UV‐C irradiation [105, 106, 107, 108]. Although none should be considered substitutes for appropriate culture‐guided antimicrobial therapy in vision‐threatening infections, they may serve as valuable components of a multimodal stewardship strategy aimed at preserving antimicrobial effectiveness while optimizing clinical outcomes.

## 9 Conclusion

Antimicrobial resistance in veterinary ophthalmology is no longer a theoretical concern; it is a clinically significant and evolving reality, particularly in canine keratitis, where multidrug resistance is now common in referral populations. Recent topical antimicrobial exposure remains the most consistent predictor of in vitro nonsusceptibility, highlighting the importance of early culture and judicious empiric therapy. Antimicrobial susceptibility testing is indispensable, yet it must be interpreted in the context of the pharmacologic realities of topical therapy and be integrated with ulcer severity, organism identity, and early clinical response.

Sustaining antimicrobial effectiveness will require coordinated efforts to develop ocular‐specific interpretive frameworks, maintain local surveillance, strengthen infection control practices, and embed stewardship into daily ophthalmic workflows. At the same time, antibiotic‐sparing adjuncts should be evaluated not as replacements for rational therapy, but as tools that may help reduce cumulative antimicrobial exposure. The goal is not simply to resolve the current infection but to preserve therapeutic reliability for future patients.

## Author Contributions

**Oren Pe'er:** writing – original draft, writing – review and editing. **Lionel Sebbag:** conceptualization, writing – original draft, writing – review and editing, project administration, investigation.

## Artificial Intelligence Statement

Generative AI tools, including ChatGPT and Gemini, were used solely for language editing and improving the clarity of the manuscript. All scientific content, interpretations, and conclusions were developed and reviewed by the authors.

## Ethics Statement

This study is a review of previously published literature; as such, ethical approval and informed consent were not required.

## Conflicts of Interest

The authors declare no conflicts of interest.

## Data Availability

Data sharing not applicable to this article as no datasets were generated or analyzed during the current study.
